# Vitamin D status, sleep patterns, genetic susceptibility, and the risk of incident adult-onset asthma: a large prospective cohort study

**DOI:** 10.3389/fnut.2023.1222499

**Published:** 2023-06-30

**Authors:** Qinyu Chang, Yiqun Zhu, Guowei Zhou, Huaying Liang, Dianwu Li, Jun Cheng, Pinhua Pan, Yan Zhang

**Affiliations:** ^1^Center of Respiratory Medicine, Xiangya Hospital, Central South University, Changsha, Hunan, China; ^2^National Key Clinical Specialty, Branch of National Clinical Research Center for Respiratory Disease, Xiangya Hospital, Central South University, Changsha, China; ^3^Hunan Engineering Research Center for Intelligent Diagnosis and Treatment of Respiratory Disease, Changsha, Hunan, China; ^4^Department of Dermatology, Xiangya Hospital, Central South University, Changsha, Hunan, China; ^5^Department of Spine Surgery, The Third Xiangya Hospital, Central South University, Changsha, Hunan, China; ^6^Department of Experimental Radiation Oncology, The University of Texas MD Anderson Cancer Center, Houston, TX, United States; ^7^National Clinical Research Center for Geriatric Disorders, Xiangya Hospital, Central South University, Changsha, China

**Keywords:** Vitamin D, sleep behaviors, genetic susceptibility, asthma, UK biobank

## Abstract

**Introduction:**

Vitamin D has been known to be associated with asthma, particularly in children, while the evidence among adults is limited and inconclusive. This study aimed to investigate the association between serum, vitamin D concentrations, and the incidence of adult-onset asthma and also the modified effect caused by sleep patterns and genetic risks.

**Methods:**

A prospective cohort study with 307,872 participants aged between 37 and 73 years was conducted based on the UK Biobank, with a median follow-up of 12 years. The Cox proportional hazard model was applied to evaluate the association between vitamin D status and incident adult-onset asthma, and the modified effect was investigated by conducting stratified analysis according to sleep pattern score and genetic risk score, and subgroup analyses were performed by sex, age, BMI, and smoking status as well.

**Results:**

Individuals with optimal vitamin D concentration were associated with 11.1% reduced risk of incident asthma compared to those participants with deficient vitamin D (HR = 0.889; 95% CI: 0.820–0.964; *p* = 0.005). Moreover, stratification analysis demonstrated that the protective effect of vitamin D on asthma risk was modified by sleep patterns or genetic susceptibility, with the strongest protective effect being observed in the subpopulation with a moderate sleep pattern (HR = 0.883; 95% CI: 0.797–0.977; *p* = 0.016) and a moderate genetic risk (HR = 0.817; 95% CI: 0.711–0.938; *p* = 0.004). In subgroup analyses, the protective effect of optimal vitamin D levels was only significant among men, individuals younger than 60 years of age, overweight individuals, and current or previous smokers.

**Conclusion:**

Increased serum vitamin D levels were associated with a lower risk of incident adult-onset asthma, and this association was modified by sleep patterns and genetic predisposition to some extent.

## 1. Introduction

As one of the most widespread, non-communicable, chronic disorders, asthma imposes a substantial global burden of disease due to lifelong treatment (1). In 2017, the global prevalence of asthma was 3.57% with 0.91% for all-cause disability-adjusted life-years (DALYs) (2). Therefore, it is very critical to take effective measures for early prevention.

Although vitamin D has been commonly recognized to regulate the immune response and inhibit a variety of inflammatory pathways (3), observational studies have not been consistent about whether vitamin D concentrations had an impact on the prevalence or incidence of asthma (4, 5). Most studies focus on the effect of vitamin D on the primary prevention of asthma among children, but studies on adult-onset asthma are scarce and inconclusive with a very small sample size (6, 7), which is required to be verified by clinical trials or large prospective studies.

As a typical chronic disease, the risk of asthma is closely related to lifestyle. Recent research studies indicate that improving sleep behaviors might slow the development and exacerbation of asthma (8, 9). Moreover, sleep duration and quality could be affected in both children and adult asthma patients, and this effect was independent of nocturnal awakening (10, 11). Meanwhile, as increasing genetic loci associated with asthma have been ascertained in genome-wide association studies (12, 13), the role of genetic susceptibility in asthma could not be underestimated. The proportion of asthma incidence attributed to heritability was limited owing to the environmental factors (14), and it had not been explicitly quantified.

Additionally, various studies provided evidence linking sleep behaviors to vitamin D metabolism. Researchers have reported that serum vitamin D concentrations may alter sleep behavior by affecting inflammatory substances (15). For example, a meta-analysis presented that individuals with vitamin D deficiency were observably related to sleep disorders, such as shorter sleep duration, poorer sleep quality, and more daytime sleepiness (16). Those participants with excessive daytime sleepiness were more likely to have low serum vitamin D levels (17). Therefore, sleep behavior might confound the association of vitamin D with adult-onset asthma.

To address the shortcomings of existing research studies, this large prospective cohort study aimed to elucidate the relationship between serum vitamin D concentrations and the incidence of adult-onset asthma using the data from the UK biobank and examine the modification effect of sleep patterns and genetic risk on this relationship as well.

## 2. Methods

### 2.1. Study design and participants

The UK Biobank is initiated by the UK government to collect and store long-term human biological samples including physiological, pathological, and socioeconomic information of the sample subjects through standardized procedures. It has recruited more than half a million participants aged 37 to 73 years from across the UK between 2006 and 2010, and the UK Biobank website was opened to public registration in 2012. This prospective cohort study was conducted based on data from the UK Biobank. All participants provided electronically signed consent forms. The North-West Multicenter Research Ethics Committee approved the study. We submitted an application to the UK Biobank database and obtained access to some of the data (application number 84979).

Our study's exclusion criteria are as follows: (1) participants with asthma at the baseline; (2) participants with missing data on the serum vitamin D concentration, sleep behavior, and genetic risk; (3) participants whose sex inferred from the relative intensity of markers on the X and Y chromosomes do not match to self-reported sex; and (4) non-white participants.

### 2.2. Assessment of serum vitamin D concentrations

The UK Biobank measured biochemical markers in blood samples from all 500,000 participants. Serum vitamin D concentration was measured by a clinical chemistry analyzer, Beckman Coulter DXI 800, using a direct competitive chemiluminescent immunoassay. The observed reportable range of serum vitamin D concentration was 10–375 nmol/L. The UK Biobank website provides more detailed information on the measurements. Based on clinical guidelines (18) and previous literature (19, 20), we defined vitamin D deficient, insufficient, and optimal as < 25, 25–50, and >50 nmol/L, respectively.

### 2.3. Definition of sleep behaviors

The sleep pattern score was established according to the previous studies (21–23). We defined the following criteria for each healthy sleep behavior: no excessive daytime sleepiness (“sometimes” or “rarely/never”); reported sometimes or rarely/never insomnia symptoms; early chronotype (“morning” or “morning than evening”); no self-reported snoring; and sleep for 7–8 h/day. If each sleep behavior meets the criteria, the score is 1; otherwise, the score is 0. The total score of five items is the score of the sleep pattern. Participants with a sleep pattern score of 4 or 5 were assigned to the “healthy sleep pattern” group, those with a score of 2 or 3 were assigned to the “intermediate sleep pattern” group, and those with a score of 1 or 0 were defined as the “poor sleep pattern” group.

### 2.4. Definition of genetic risk score

The UK Biobank collected genetic data from 488,377 participants and determined it with two very similar genotyping arrays to capture genome-wide genetic variation, including short insertions and deletions (indels) and single nucleotide polymorphism (SNPs) (24). According to the large and comprehensive genome-wide analysis by Yi Han et al., we select 212 independent SNPs associated with asthma to construct the weighted genetic risk scores (GRS). Each GRS was weighted by the overall effect sizes of the included alleles from the association analysis including both sexes (12) (Supplementary Table 1). Participants were divided into three groups (high, intermediate, and low genetic risk groups) according to tertiles of the genetic risk score.

### 2.5. Assessment of outcomes

Asthma was defined with the International Classification Disease (ICD-10) code (J45) with all sources of asthma, including self-report, hospital admission data, primary care, and death register in the national hospital registers. Individuals were followed until the date of occurrence of asthma, the end of follow-up in December 2020, or death, whichever came first. Moreover, death registry records were used to ascertain the death.

### 2.6. Statistical analysis

We used number (proportion) and mean (standard deviation) to describe categorical variables and normally distributed continuous variables of recruited individuals, respectively. The comparison between groups was evaluated by chi-square tests or student *t*-test, and the association of serum vitamin D (categorized by clinical cut-offs) with the incidence of asthma was evaluated by the Cox proportional hazard model. All variables met the proportional hazards assumption, and the categorical variables were tested by Kaplan–Meier estimation and the continuous variables by the Schoenfeld residual method. We then stratified the association between serum vitamin D and asthma incidence according to the overall sleep pattern score and genetic risk. A subgroup analysis based on sex, age, BMI, and smoking status was also conducted. Furthermore, the relative excess risk (RERI) and the attributable proportion (AP) were calculated by a SAS program to estimate the additive interaction effect between vitamin D and sleep patterns or genetic risk. In addition, the population-attributable fraction (PAF) was calculated to compare the changes in the incidence rate after altering a specific exposure. To ensure the stability of our results, we conducted sensitivity analysis from the following two aspects: (1) excluding participants with asthma within 3 years of enrollment to reduce the possibility of reverse causality; (2) performing multiple imputations for missing covariates, including BMI, income, education, smoking status, and vitamin D supplement.

## 3. Results

### 3.1. Baseline characteristics of the individuals recruited

After excluding participants based on the criteria described in the method, a total of 307,872 participants were recruited for the main analysis (Supplementary Figure 1), with an asthma prevalence of 2.0% (n = 6,143). The median follow-up was 12 years for the entire cohort and 5 years for individuals with asthma. The baseline characteristics of the individuals were described in Supplementary Table 2 based on the vitamin D clinical cutoffs. Evidently, with the increase in serum vitamin D concentrations, the incidence of asthma gradually decreases. People with older age, lower BMI, as well as higher income, tended to have increased vitamin D levels. Furthermore, many of them were taking vitamin D regularly, and few of them were current smokers. In terms of sleep behaviors, individuals with higher vitamin D concentrations had higher overall sleep pattern scores, which means healthier sleep behavior.

### 3.2. Association of vitamin D levels with adult-onset incident asthma

The association between serum vitamin D concentrations and the incidence of asthma was represented in Table 1. In all three models, serum vitamin D levels were inversely linked with incident asthma. Among individuals with optimal vitamin D concentrations, the hazard ratios of asthma were reduced by 11.1% (HR = 0.889; 95% CI: 0.820–0.964; *p* = 0.005) in the full-adjusted model, including age, sex, race, BMI, income, education, smoking status, assessment center, and vitamin D supplements, which indicated the robust protective role of optimal vitamin D on preventing the incidence of asthma. Sensitivity analysis was conducted after excluding participants with asthma within 3 years and performing multiple imputations for missing covariates and yielded similar results (Supplementary Tables 3, 4).

**Table 1:** Associations of the risk for adult-onset incident asthma and serum vitamin D concentrations.

**Vitamin D concentration, nmol/L**	**Case/N**	**Model 1** ^**a**^		**Model 2** ^**b**^		**Model 3** ^**c**^	
		**HR (95% CI)**	***P***	**HR (95% CI)**	***P***	**HR (95% CI)**	***P***
**Category**							
Deficient (< 25)	808/35,495	Ref.	–	Ref.	–	Ref.	–
Insufficient (25–50)	2,629/127,446	0.905 (0.837, 0.980)	0.014	0.887 (0.820, 0.960)	0.003	0.944 (0.871, 1.022)	0.154
Optimal (>50)	2,706/144,931	0.819 (0.757, 0.886)	< 0.001	0.786 (0.726, 0.851)	< 0.001	0.889 (0.820, 0.964)	0.005
P_trend_		< 0.001		< 0.001		0.009	

^a^Model 1: Crude.

^b^Model 2: Adjusted for age, sex, and race.

^c^Model 3: Adjusted for age, sex, race, BMI, income, education, smoking status, assessment center, and vitamin D supplements.

HR, Hazard Ratio; CI, Confidence Interval.

### 3.3. Subgroup analyses of relationships between serum vitamin D concentrations and incident asthma

**Figure 1:**
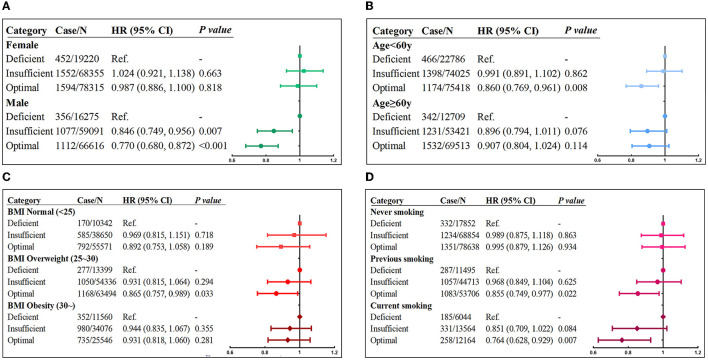
Risk of incident asthma associated with serum vitamin D concentrations stratified by **(A)** sex, **(B)** age, **(C**) BMI, and **(D)** smoking status. Adjusted for age, sex, race, BMI, income, education, smoking status, assessment center, and vitamin D supplements.

Subgroup analyses based on sex indicated that the relationship between serum vitamin D levels and incident asthma was only statistically significant in men (Figure 1A) but not in women. Men with optimal vitamin D concentration had 23.0% (HR = 0.770; 95% CI: 0.680–0.872; *p* < 0.001) lower risk of asthma. As age subgroup analysis manifested (Figure 1B), optimal vitamin D concentration was a protective factor for preventing asthma, and the impact size was stronger in people younger than 60 years (HR = 0.860; 95% CI: 0.769–0.961; *p* = 0.008) but not in those older than 60 years. The results of the BMI subgroup (Figure 1C) showed that the protective effect of optimal serum vitamin D on asthma was the most obvious in overweight individuals (HR = 0.865; 95% CI: 0.757–0.989; *p* = 0.033). In the smoking subgroup analysis (Figure 1D), optimal vitamin D levels had the strongest protective effect among current smokers (HR = 0.764; 95% CI: 0.628–0.929; *p* = 0.007), followed by previous smokers (HR = 0.855; 95% CI: 0.749–0.977; *p* = 0.022), which was not observed among never-smokers.

### 3.4. Relationships between serum vitamin D concentrations and incident asthma stratified by sleep behaviors and genetic risk

**Figure 2:**
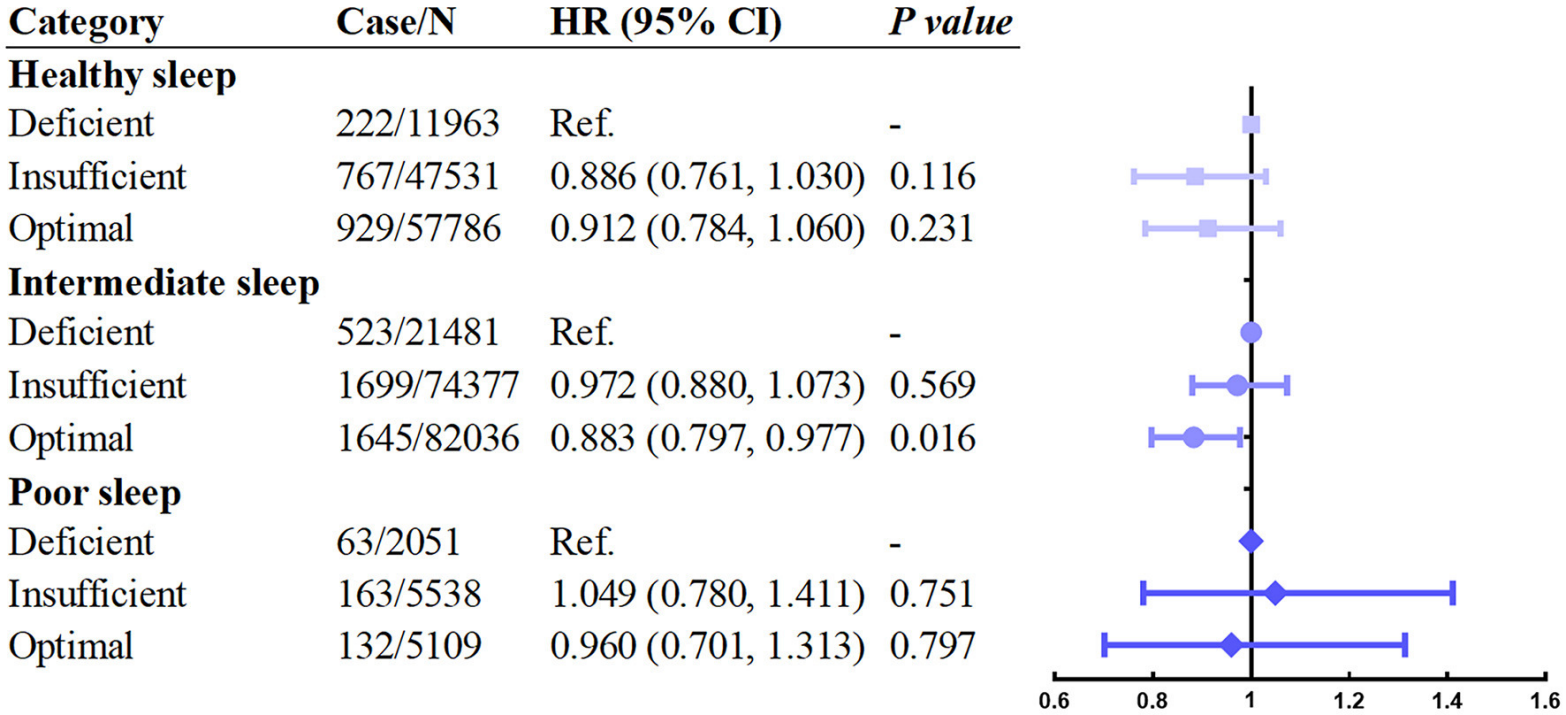
Association between serum vitamin D concentrations and asthma stratified by overall sleep patterns. Adjusted for age, sex, race, BMI, income, education, smoking status, assessment center, and vitamin D supplements.

**Figure 3:**
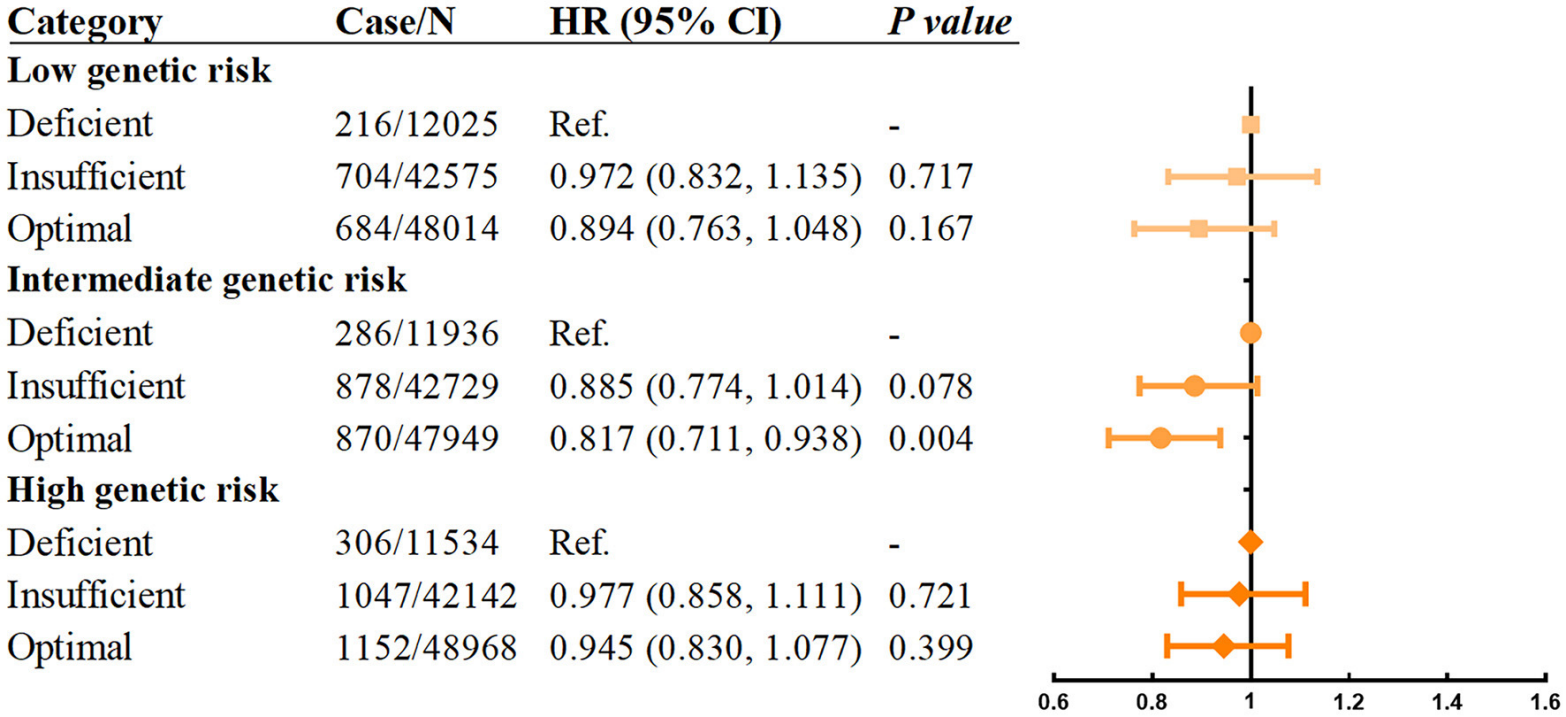
Association between serum vitamin D concentrations and asthma stratified by genetic risk. Adjusted for age, sex, race, BMI, income, education, smoking status, assessment center, and vitamin D supplements.

Next, we investigated whether the relationship between vitamin D levels with incident asthma was modified by sleep behaviors and genetic risk. The results showed that individuals with higher sleep pattern scores represented a healthier sleep pattern and had a significantly decreased incidence of asthma (Figure 2). A prominent interaction between vitamin D levels and sleep behaviors on incident asthma was observed in stratified analysis based on the overall sleep patterns. The protective hazard ratio of higher vitamin D levels was only observed in intermediate sleep groups with the HR (95% CI) of 0.883 (0.797–0.977) in participants with optimal vitamin D levels compared to deficiency vitamin D levels. According to the stratified analysis of the GRS of asthma, the effect was similar, and the incidence of asthma increased when the genetic risk increased (Figure 3). Only in participants with intermediate genetic risk, the protective effect was significant (HR = 0. 817; 95% CI: 0.711–0.938; *p* = 0.004).

### 3.5. Interaction effect and population-attributable fractions

Measures for the interaction of vitamin D concentrations with sleep patterns and genetic risk are shown in Table 2. RERI and AP for the linkage between insufficient vitamin D concentrations and intermediate sleep pattern were 0.146 (0.024–0.267) and 0.113 (0.018–0.209) compared to optimal vitamin D concentrations and healthy sleep pattern, which means the linkage was responsible for 0.146 relative excess risks and 13% of the total risk of asthma. The results manifested a positive additive interaction between insufficient vitamin D concentrations and intermediate sleep patterns. In addition, the multiplicative interactions were significant between vitamin D concentrations and sleep behaviors or genetic risk (Supplementary Table 5). According to the PAF per group (Table 3), we discovered that genetic susceptibility had the strongest influence on the risk of asthma, with 60.1% (95% CI: 0.579–0.621) of incident asthma attributable to non-low genetic risk, 26.2% (95% CI: 0.222–0.301) attributable to a non-ideal sleep pattern, and 11.5% (95% CI: 0.070–0.158) attributable to inappropriate vitamin D concentration.

**Table 2:** Additive interaction of serum vitamin D levels with genetic risk and sleep patterns.

	**Insufficient**		**Deficient**	
	**RERI (95% CI)**	**AP (95% CI)**	**RERI (95% CI)**	**AP (95% CI)**
**Sleep patterns**				
Intermediate sleep	0.146 (0.024, 0.267)	0.113 (0.018, 0.209)	0.083 (−0.123, 0.289)	0.061 (−0.088, 0.209)
Poor sleep	0.182 (−0.168, 0.534)	0.118 (−0.098, 0.333)	0.061 (−0.429, 0.550)	0.038 (−0.263, 0.340)
**Genetic risk**				
Intermediate genetic risk	−0.010 (−0.177, 0.156)	−0.008 (−0.130, 0.115)	0.154 (−0.112, 0.421)	0.096 (−0.064, 0.257)
High genetic risk	−0.085 (−0.266, 0.097)	−0.050 (−0.158, 0.058)	−0.079 (−0.362, 0.204)	−0.045 (−0.209, 0.119)

To estimate RERI and AP, references were optimal vitamin D concentrations, low genetic risk, and healthy sleep pattern.

Models for genetic risk and sleep patterns were adjusted for age, sex, race, BMI, income, education, smoking status, assessment center, and vitamin D supplements.

AP, Attributable proportion due to interaction; RERI, relative excess risk due to interaction; CI, Confidence Interval.

**Table 3:** Population-attributable fraction per group.

	**Groups**	**PAF (%) (95% CI)**	***P***
Serum VD level	Non-optimal to optimal serum VD level	11.5 (0.070–0.158)	< 0.001
	Deficient to optimal serum VD level	18.0 (0.114–0.241)	< 0.001
	Insufficient to optimal serum VD level	9.5 (0.045–0.142)	0.002
Sleep pattern	Non-healthy to healthy sleep pattern	26.2 (0.222–0.301)	< 0.001
	Intermediate to healthy sleep pattern	24.8 (0.206–0.287)	< 0.001
	Poor to healthy sleep pattern	42.0 (0.352–0.481)	< 0.001
Genetic risk	Non-low to low genetic risk	60.1 (0.579–0.621)	< 0.001
	Intermediate to low genetic risk	21.1 (0.159–0.261)	< 0.001
	High to low genetic risk	73.0 (0.714–0.744)	< 0.001

PAF, population-attributable fraction; CI, Confidence Interval; VD, Vitamin D.

## 4. Discussion

In our large prospective cohort based on the UK Biobank, a significant relationship between increased serum vitamin D levels and decreased risk of incident adult-onset asthma was revealed. These association was modified by sleep patterns and genetic predisposition and was relatively robust in subgroup populations with intermediate sleep patterns and intermediate genetic risks.

The association between serum vitamin D concentrations and the risk of adult-onset asthma was scarce and currently inconclusive. Two cross-sectional studies of adults from Canada and the United States have indicated that serum vitamin D levels below 50 or 30 nmol/L were related to an increased odds ratio of current asthma (25, 26). In contrast, a Korean study based on 15,212 adult Asian individuals concluded that adults with vitamin D below 20 nmol/L were not associated with an increased prevalence of asthma (27). Meanwhile, a large Israeli adult study including 308,000 members also suggested that vitamin D status was not significantly associated with a diagnosis of asthma but rather with acute exacerbations of asthma (28). These differences were likely due to the characteristics of the study populations or might be regional. Our recent cross-sectional study based on a large population not only verified the linkage between optimal vitamin D (>50 nmol/L) and decreased prevalence of asthma but also discovered that vitamin D deficiency (< 25 nmol/L) was associated with poor asthma control (29). However, due to the limitation of the cross-sectional study, this large prospective cohort study based on the UK Biobank was conducted to further support the protective role of vitamin D and reduced risk of adult-onset asthma.

Surprisingly, in this study, we observed a protective role of serum vitamin D concentrations on the incidence of adult-onset asthma, particularly in men rather than women. A similar finding has been reported in a Norwegian HUNT study, which manifested that low vitamin D concentrations were related to the high risk of asthma only in men without allergic rhinitis (30). Epidemiological studies manifested that the incidence and severity of asthma in women were more unstable (31, 32). This discrepancy might be attributed to the differences in lung anatomy or the effect of sex hormones (33, 34). On the other hand, the protective effect of vitamin D on adult-onset asthma was stronger in people younger than 60 years, which was first identified. This result might be explained by the promoting effect of vitamin D on aging-related pulmonary fibrosis (35, 36). The protective effect of vitamin D was more likely to be observed among overweight or obese individuals because of the higher incidence of asthma. However, in obese individuals, the bioavailability of vitamin D from cutaneous and dietary sources is reduced because it is deposited in body fat (37), which partially offset the protective effect of vitamin D. Moreover, animal experiments have demonstrated that low doses of vitamin D could effectively protect the lungs of the mouse from cigarette smoke-induced damage (38). Therefore, the protective effect of serum vitamin D levels on asthma was more obvious in current or previous smokers than in never smokers.

Furthermore, the effect of sleep patterns and genetic risk on asthma risk might partially explain the uncertainty about the relationship between serum vitamin D concentrations and incident asthma. The protective effect of vitamin D was only seen in individuals who were not affected by sleep patterns and genetic predisposition, that is, those at the intermediate risk. The relatively small sample size of the healthy and poor sleep pattern groups could be one of the reasons. In addition, the overall sleep pattern had a complex interaction with vitamin D function and metabolism, as well as the risk of asthma. Poor sleep patterns, such as daytime sleepiness, might be associated with less outdoor activity and sun exposure time, which affected vitamin D synthesis of the skin (39); insomnia and irregular chronotype were closely related to mental disorders such as depression (40), which might also be due to poor outdoor activity and sun exposure time; snoring might be associated with abnormal airway structure, and it was possible to increase the risk of asthma (41); either too long or short sleep duration might be associated with abnormal melatonin secretion (42), a competing binding molecule of the vitamin D receptor (43). The instability of asthma incidence in the population with healthy or poor sleep patterns might also account for its lack of association with serum vitamin D levels.

In the stratified analysis of genetic susceptibility, although the protective effect of optimal vitamin D level was statistically significant only in the intermediate genetic risk group, the risk of asthma in each group showed a decreasing trend with the increase in the serum vitamin D level, indicating that the protective effect of vitamin D was limited in people with high or low genetic susceptibility to asthma. Numerous research studies identified a critical role of genetic susceptibility in asthma development (44). Therefore, the influence of acquired environment or living habits and nutrition on the pathogenesis of asthma might be overridden by genetic factors.

### 4.1. Strengths and limitations

Compared with previous studies, this study has certain advantages, it is a prospective large sample size cohort study with a median follow-up of 12 years. In sensitivity analyses, we excluded patients with asthma developed within the first 3 years of enrolment, reducing the possibility of reverse causality. Certainly, several limitations are found in this study. First, the participants included were between 37 and 73 years of age and were restricted to European ancestry; thus, the results had the limitation of extrapolating them into adolescents, young adults, or other races. Second, because not all individuals participated in the follow-up and specific follow-up time was not reported, the evaluation of vitamin D level and sleep behavior were at the baseline and not longitudinally assessed throughout the study process. Hence, our study still could not prove a causal relationship between serum vitamin D levels and asthma.

## 5. Conclusion

Our study discovered that optimal serum vitamin D concentrations were associated with a reduced risk of incident adult-onset asthma, which was significant in men, individuals younger than 60 years of age, overweight individuals, and current or previous smokers. Additionally, to some extent, this association was modified by sleep patterns or genetic predisposition. Collectively, our findings support the protective role of vitamin D on reduced risk of adult-onset asthma.

## Data availability statement

The datasets presented in this study can be found in online repositories. The names of the repository/repositories and accession number(s) can be found in the article/Supplementary material.

## Ethics statement

The studies involving human participants were reviewed and approved by North West Multicenter Research Ethics Committee (Application number 84979). The patients/participants provided their written informed consent to participate in this study.

## Author contributions

YZha developed the concept and provided data sources. PP is responsible for supervision and management. QC, YZhu, and GZ completed the methodology and writing. HL, DL, and JC performed the investigation and validation. All authors read and approved the final manuscript.
