# Supplementary material for: Vitamin D status, sleep patterns, genetic susceptibility, and the risk of incident adult-onset asthma: a large prospective cohort study
Source: Front Nutr. 2023 Jun 30;10:1222499. doi: 10.3389/fnut.2023.1222499 (PMC10349527; doi:10.3389/fnut.2023.1222499)
Supplement: Supplementary file 1 [file Data_Sheet_1.docx]

Supplementary Material

Vitamin D status, sleep patterns, genetic susceptibility, and the risk of incident adult-onset asthma: A large prospective cohort study

Qinyu Chang1,2,3†, Yiqun Zhu1,2,3†, Guowei Zhou4†, Huaying Liang1,2,3, Dianwu Li1,2,3, Jun Cheng5,6, Pinhua Pan1,2,3,7*, Yan Zhang1,2,3,7*

*** Correspondence:**

Yan Zhang, Center of Respiratory Medicine, Xiangya Hospital, Central South University, Changsha 410008, Hunan, China. [zhangy4290@csu.edu.cn](mailto:zhangy4290@csu.edu.cn) (ORCiD: 0000-0003-0246-2965)

Pinhua Pan, Center of Respiratory Medicine, Xiangya Hospital, Central South University, Changsha 410008, Hunan, China. [pinhuapan668@csu.edu.cn](mailto:pinhuapan668@csu.edu.cn) (ORCiD: 0000-0001-5883-0531)

# Supplementary Figures


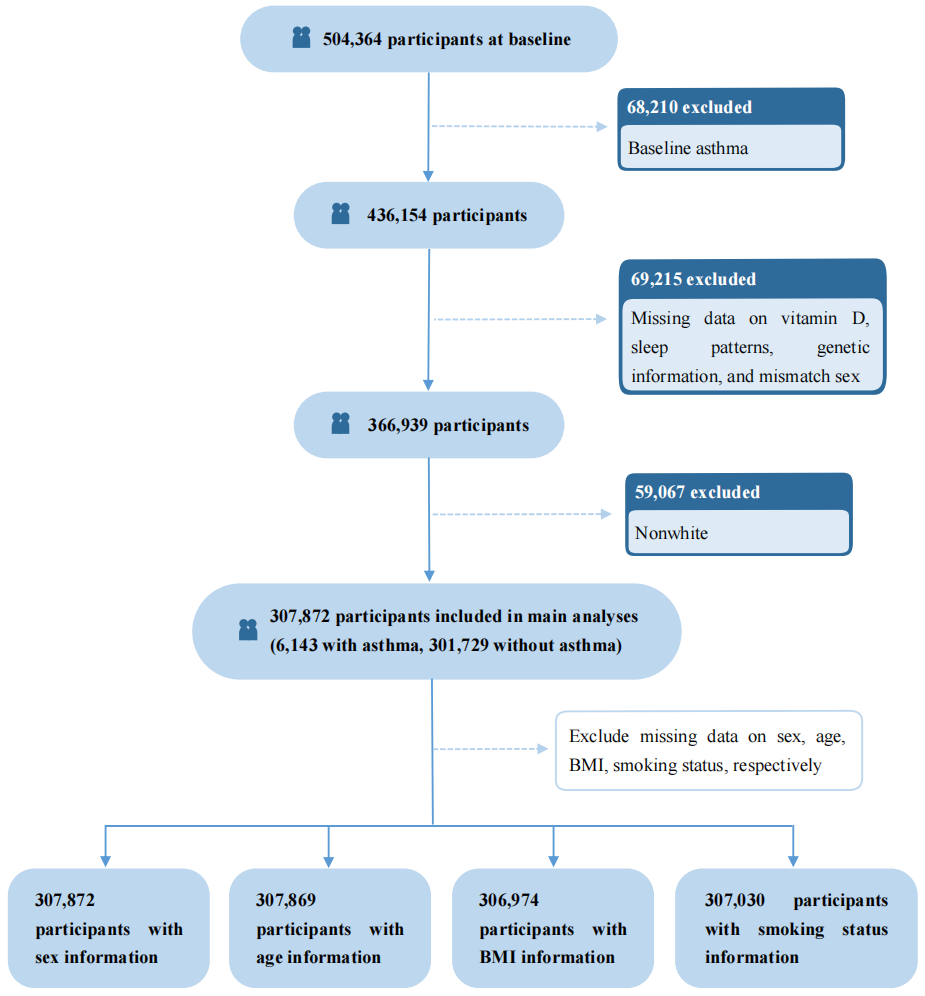


**Figure S1. Flow diagram of eligible population selection from the UK Biobank Study.**

# Supplementary Tables

**Table S1. Single-nucleotide polymorphisms used to build the genetic risk score for asthma.**

|  |  |  |  |  | **Yi Han et al, 2020, 32296059** | | |
| --- | --- | --- | --- | --- | --- | --- | --- |
|  | **SNP** | **CHR** | **BP** | **EA** | **Beta** | **SE** | ***P*** |
| 1 | rs734999 | 1p36.32 | 2,513,216 | C | 0.04 | 0.0060 | 4.0E-11 |
| 2 | rs301819 | 1p36.23 | 8,501,786 | G | 0.05 | 0.0061 | 1.2E-13 |
| 3 | rs6687430 | 1p36.22 | 10,633,245 | A | 0.04 | 0.0060 | 7.2E-11 |
| 4 | rs2230624 | 1p36.22 | 12,175,658 | G | 0.18 | 0.0253 | 4.3E-12 |
| 5 | rs1293202 | 1p36.11 | 25,263,997 | G | 0.06 | 0.0092 | 1.6E-11 |
| 6 | rs11577318 | 1p36.11 | 26,601,570 | G | 0.03 | 0.0077 | 4.8E-05 |
| 7 | rs2228552 | 1p35.2 | 32,165,495 | T | 0.03 | 0.0062 | 3.4E-08 |
| 8 | rs648973 | 1p34.3 | 36,518,627 | A | 0.08 | 0.0273 | 4.6E-03 |
| 9 | rs7542867 | 1p22.1 | 93,076,518 | A | 0.03 | 0.0062 | 2.5E-06 |
| 10 | rs71580314 | 1q21.3 | 150,507,691 | A | 0.04 | 0.0060 | 1.6E-09 |
| 11 | rs61816761 | 1q21.3 | 152,285,861 | A | 0.29 | 0.0219 | 3.5E-39 |
| 12 | rs4129267 | 1q21.3 | 154,426,264 | T | 0.04 | 0.0061 | 1.4E-11 |
| 13 | rs3027001 | 1q23.2 | 159,169,463 | C | 0.04 | 0.0071 | 5.5E-08 |
| 14 | rs2070901 | 1q23.3 | 161,185,058 | T | 0.05 | 0.0067 | 3.6E-12 |
| 15 | rs1214598 | 1q24.2 | 167,426,424 | G | 0.06 | 0.0062 | 2.9E-21 |
| 16 | rs10912564 | 1q25.1 | 173,170,618 | T | 0.05 | 0.0065 | 1.5E-14 |
| 17 | rs17669032 | 1q31.3 | 198,653,174 | A | 0.07 | 0.0099 | 8.6E-13 |
| 18 | rs7555556 | 1q32.1 | 203,090,976 | C | 0.04 | 0.0064 | 3.0E-11 |
| 19 | rs4127124 | 1q32.1 | 206,624,362 | A | 0.04 | 0.0060 | 4.6E-09 |
| 20 | rs906364 | 1q32.3 | 212,858,748 | C | 0.04 | 0.0078 | 1.4E-07 |
| 21 | rs697852 | 1q42.12 | 226,914,734 | A | 0.05 | 0.0079 | 1.5E-09 |
| 22 | rs10178845 | 2p25.1 | 8,443,803 | G | 0.07 | 0.0066 | 1.7E-24 |
| 23 | rs72787718 | 2p23.1 | 30,474,351 | A | 0.04 | 0.0071 | 3.2E-08 |
| 24 | rs68110799 | 2p16.1 | 60,945,555 | A | 0.04 | 0.0069 | 8.9E-08 |
| 25 | rs13421997 | 2p14 | 64,946,639 | G | 0.03 | 0.0068 | 8.0E-07 |
| 26 | rs35548551 | 2p13.2 | 72,039,714 | T | 0.05 | 0.0092 | 4.4E-07 |
| 27 | rs3771180 | 2q12.1 | 102,953,617 | G | 0.17 | 0.0086 | 2.1E-92 |
| 28 | rs13405741 | 2q13 | 111,913,056 | C | 0.05 | 0.0102 | 2.4E-07 |
| 29 | rs2723197 | 2q13 | 113,689,747 | G | 0.03 | 0.0061 | 2.4E-06 |
| 30 | rs7425446 | 2q22.2 | 143,777,498 | A | 0.04 | 0.0076 | 2.2E-08 |
| 31 | rs6741949 | 2q24.2 | 162,910,223 | G | 0.03 | 0.0061 | 1.0E-08 |
| 32 | rs2595389 | 2q32.1 | 187,534,183 | T | 0.03 | 0.0060 | 1.0E-05 |
| 33 | rs2164068 | 2q33.1 | 198,943,852 | T | 0.03 | 0.0060 | 5.4E-08 |
| 34 | rs13383994 | 2q36.1 | 224,667,692 | A | 0.03 | 0.0061 | 5.3E-06 |
| 35 | rs7423358 | 2q36.3 | 228,704,721 | C | 0.05 | 0.0070 | 2.2E-12 |
| 36 | rs9247 | 2q37.1 | 234,113,301 | C | 0.03 | 0.0076 | 5.9E-04 |
| 37 | rs34290285 | 2q37.3 | 242,698,640 | G | 0.12 | 0.0068 | 1.7E-70 |
| 38 | rs334782 | 3p26.2 | 3,148,458 | C | 0.03 | 0.0062 | 1.7E-05 |
| 39 | rs13101202 | 3p25.2 | 12,699,361 | C | 0.05 | 0.0080 | 2.1E-09 |
| 40 | rs2014490 | 3p24.3 | 16,972,211 | G | 0.04 | 0.0063 | 9.2E-10 |
| 41 | rs2036226 | 3p24.3 | 23,597,986 | T | 0.03 | 0.0061 | 7.5E-08 |
| 42 | rs35570272 | 3p22.3 | 33,047,662 | T | 0.06 | 0.0062 | 1.5E-21 |
| 43 | rs73072483 | 3p21.2 | 50,771,624 | G | 0.05 | 0.0090 | 1.4E-07 |
| 44 | rs9813229 | 3p14.3 | 56,741,585 | A | 0.03 | 0.0062 | 1.4E-07 |
| 45 | rs7372960 | 3p13 | 71,254,751 | T | 0.04 | 0.0070 | 1.4E-07 |
| 46 | rs61192126 | 3p13 | 72,394,852 | T | 0.04 | 0.0066 | 6.9E-10 |
| 47 | rs55749605 | 3q12.3 | 101,232,093 | A | 0.03 | 0.0062 | 3.2E-05 |
| 48 | rs7622814 | 3q13.2 | 112,650,431 | T | 0.03 | 0.0060 | 9.8E-09 |
| 49 | rs9877891 | 3q13.33 | 119,260,866 | C | 0.04 | 0.0078 | 2.7E-08 |
| 50 | rs10049074 | 3q13.33 | 121,702,325 | T | 0.03 | 0.0060 | 6.9E-08 |
| 51 | rs2811518 | 3q21.3 | 128,013,645 | A | 0.05 | 0.0092 | 1.1E-08 |
| 52 | rs13077048 | 3q23 | 141,106,954 | T | 0.04 | 0.0061 | 2.5E-10 |
| 53 | rs17485347 | 3q26.2 | 169,127,519 | C | 0.03 | 0.0069 | 2.3E-06 |
| 54 | rs7626218 | 3q26.32 | 176,852,038 | A | 0.05 | 0.0061 | 4.5E-15 |
| 55 | rs13099273 | 3q28 | 188,133,518 | A | 0.06 | 0.0061 | 2.5E-22 |
| 56 | rs7636495 | 3q29 | 196,367,936 | G | 0.06 | 0.0079 | 1.4E-12 |
| 57 | rs9291151 | 4p16.2 | 4,775,137 | T | 0.03 | 0.0061 | 6.0E-07 |
| 58 | rs5743618 | 4p14 | 38,798,648 | C | 0.10 | 0.0073 | 6.8E-43 |
| 59 | rs73242688 | 4q12 | 58,236,911 | C | 0.08 | 0.0209 | 1.1E-04 |
| 60 | rs227361 | 4q24 | 103,586,977 | T | 0.03 | 0.0060 | 8.4E-07 |
| 61 | rs34712979 | 4q24 | 106,819,053 | A | 0.04 | 0.0070 | 3.5E-07 |
| 62 | rs4145717 | 4q27 | 123,316,076 | T | 0.07 | 0.0064 | 3.9E-24 |
| 63 | rs111379736 | 4q31.21 | 143,763,739 | A | 0.02 | 0.0065 | 3.7E-03 |
| 64 | rs6842889 | 4q31.21 | 145,479,880 | T | 0.03 | 0.0062 | 3.0E-06 |
| 65 | rs7734081 | 5p15.32 | 6,126,715 | C | 0.02 | 0.0060 | 1.8E-03 |
| 66 | rs16903574 | 5p15.2 | 14,610,309 | G | 0.10 | 0.0118 | 4.5E-16 |
| 67 | rs12697352 | 5p13.2 | 35,837,234 | G | 0.06 | 0.0064 | 2.2E-18 |
| 68 | rs7725052 | 5p13.1 | 40,487,270 | T | 0.03 | 0.0061 | 1.9E-05 |
| 69 | rs1501641 | 5q12.1 | 59,188,105 | A | 0.02 | 0.0062 | 7.3E-04 |
| 70 | rs10040192 | 5q13.2 | 71,695,880 | C | 0.03 | 0.0061 | 3.2E-08 |
| 71 | rs1837253 | 5q22.1 | 110,401,872 | C | 0.11 | 0.0068 | 2.7E-57 |
| 72 | rs3813308 | 5q23.1 | 118,690,781 | G | 0.04 | 0.0060 | 3.9E-11 |
| 73 | rs11950815 | 5q31.1 | 130,955,487 | G | 0.04 | 0.0063 | 8.6E-12 |
| 74 | rs848 | 5q31.1 | 131,996,500 | A | 0.10 | 0.0076 | 1.1E-39 |
| 75 | rs62379371 | 5q31.1 | 133,439,274 | G | 0.08 | 0.0142 | 6.5E-09 |
| 76 | rs10875596 | 5q31.3 | 141,495,715 | C | 0.05 | 0.0062 | 6.6E-16 |
| 77 | rs11746314 | 5q33.3 | 156,752,957 | G | 0.09 | 0.0130 | 4.2E-11 |
| 78 | rs61665417 | 5q34 | 159,918,335 | C | 0.04 | 0.0067 | 2.1E-11 |
| 79 | rs56297224 | 5q35.3 | 176,815,880 | A | 0.04 | 0.0069 | 2.4E-08 |
| 80 | rs12196306 | 6p25.3 | 249,788 | G | 0.04 | 0.0077 | 1.1E-06 |
| 81 | rs3777755 | 6p24.1 | 12,159,699 | C | 0.03 | 0.0065 | 2.1E-07 |
| 82 | rs13203384 | 6p22.3 | 19,341,301 | A | 0.04 | 0.0074 | 1.8E-06 |
| 83 | rs952579 | 6p22.3 | 21,884,440 | A | 0.05 | 0.0086 | 2.4E-09 |
| 84 | rs766406 | 6p22.2 | 26,319,588 | T | 0.04 | 0.0063 | 7.8E-09 |
| 85 | rs1233578 | 6p22.1 | 28,712,247 | G | 0.05 | 0.0078 | 3.7E-12 |
| 86 | rs9272346 | 6p21.32 | 32,604,372 | A | 0.15 | 0.0061 | 1.4E-127 |
| 87 | rs411919 | 6p21.32 | 33,194,477 | G | 0.04 | 0.0068 | 4.1E-11 |
| 88 | rs9394288 | 6p21.31 | 35,222,010 | T | 0.05 | 0.0076 | 2.3E-09 |
| 89 | rs6902766 | 6p12.3 | 51,370,323 | T | 0.04 | 0.0093 | 1.4E-04 |
| 90 | rs1504215 | 6q15 | 91,006,227 | G | 0.08 | 0.0064 | 1.3E-35 |
| 91 | rs9372120 | 6q21 | 106,667,535 | G | 0.03 | 0.0075 | 5.1E-06 |
| 92 | rs11759732 | 6q21 | 109,370,006 | A | 0.05 | 0.0084 | 1.9E-08 |
| 93 | rs813915 | 6q22.33 | 128,287,313 | T | 0.06 | 0.0070 | 3.6E-17 |
| 94 | rs4526212 | 6q23.3 | 135,804,631 | A | 0.04 | 0.0062 | 6.5E-11 |
| 95 | rs17264332 | 6q23.3 | 138,005,515 | A | 0.05 | 0.0073 | 6.4E-11 |
| 96 | rs13190880 | 6q24.2 | 143,222,770 | T | 0.06 | 0.0104 | 8.5E-08 |
| 97 | rs431362 | 6q25.1 | 149,787,378 | A | 0.04 | 0.0063 | 1.5E-08 |
| 98 | rs4599658 | 6q25.2 | 155,074,505 | G | 0.03 | 0.0061 | 1.8E-08 |
| 99 | rs895990 | 6q25.3 | 156,434,876 | G | 0.02 | 0.0061 | 1.2E-03 |
| 100 | rs62438869 | 6q27 | 167,400,917 | A | 0.04 | 0.0090 | 3.9E-06 |
| 101 | rs73033536 | 7p22.2 | 3,149,883 | T | 0.07 | 0.0106 | 8.1E-11 |
| 102 | rs10244416 | 7p21.1 | 20,575,751 | C | 0.06 | 0.0060 | 8.4E-21 |
| 103 | rs1800797 | 7p15.3 | 22,766,221 | G | 0.04 | 0.0062 | 1.1E-11 |
| 104 | rs917117 | 7p15.1 | 28,176,305 | A | 0.07 | 0.0074 | 1.9E-22 |
| 105 | rs9886239 | 7p12.2 | 50,336,551 | A | 0.03 | 0.0063 | 5.9E-06 |
| 106 | rs2190097 | 7q11.23 | 77,038,945 | C | 0.04 | 0.0071 | 3.2E-08 |
| 107 | rs10258293 | 7q22.3 | 105,662,690 | C | 0.02 | 0.0081 | 7.9E-03 |
| 108 | rs35113952 | 8p23.3 | 1,789,817 | T | 0.02 | 0.0066 | 8.7E-03 |
| 109 | rs7014953 | 8p23.1 | 8,168,413 | A | 0.03 | 0.0062 | 2.3E-07 |
| 110 | rs4739738 | 8q21.13 | 81,291,645 | G | 0.07 | 0.0062 | 2.2E-32 |
| 111 | rs12548612 | 8q23.3 | 117,349,990 | C | 0.03 | 0.0095 | 4.2E-03 |
| 112 | rs13277355 | 8q24.21 | 128,777,719 | A | 0.05 | 0.0067 | 1.3E-14 |
| 113 | rs34173062 | 8q24.3 | 145,158,607 | A | 0.09 | 0.0125 | 1.3E-12 |
| 114 | rs992969 | 9p24.1 | 6,209,697 | A | 0.13 | 0.0069 | 1.9E-78 |
| 115 | rs10960423 | 9p23 | 11,991,732 | A | 0.09 | 0.0268 | 5.9E-04 |
| 116 | rs274945 | 9p21.3 | 23,585,741 | A | 0.03 | 0.0060 | 1.2E-08 |
| 117 | rs10738765 | 9p21.2 | 27,259,224 | C | 0.03 | 0.0078 | 5.2E-04 |
| 118 | rs1537504 | 9q22.33 | 101,829,542 | A | 0.04 | 0.0071 | 1.5E-07 |
| 119 | rs4978607 | 9q32 | 117,508,437 | C | 0.05 | 0.0092 | 2.3E-08 |
| 120 | rs1930781 | 9q33.2 | 123,687,834 | G | 0.04 | 0.0063 | 7.9E-09 |
| 121 | rs10986311 | 9q33.3 | 127,071,493 | C | 0.03 | 0.0062 | 2.7E-08 |
| 122 | rs11539209 | 9q34.11 | 131,483,551 | T | 0.07 | 0.0110 | 9.7E-11 |
| 123 | rs782134971 | 9q34.2 | 136,139,907 | G | 0.03 | 0.0069 | 1.5E-05 |
| 124 | rs117137535 | 9q34.3 | 140,500,443 | A | 0.12 | 0.0205 | 3.9E-09 |
| 125 | rs4749894 | 10p15.1 | 6,058,323 | A | 0.06 | 0.0071 | 9.1E-16 |
| 126 | rs2197415 | 10p14 | 9,062,856 | G | 0.10 | 0.0061 | 5.9E-60 |
| 127 | rs9284092 | 10p11.23 | 30,801,718 | G | 0.03 | 0.0068 | 8.5E-06 |
| 128 | rs12769745 | 10q11.21 | 43,749,700 | A | 0.03 | 0.0067 | 1.5E-06 |
| 129 | rs2893907 | 10q21.2 | 64,382,359 | C | 0.03 | 0.0061 | 2.9E-08 |
| 130 | rs1134777 | 10q22.2 | 75,538,651 | C | 0.04 | 0.0068 | 2.3E-07 |
| 131 | rs113092121 | 10q23.33 | 94,384,514 | TTCTC | 0.04 | 0.0061 | 5.9E-12 |
| 132 | rs12764214 | 10q24.2 | 100,072,209 | C | 0.01 | 0.0063 | 2.5E-02 |
| 133 | rs11191385 | 10q24.32 | 104,513,049 | G | 0.03 | 0.0065 | 3.2E-05 |
| 134 | rs12788104 | 11p15.5 | 1,123,739 | G | 0.05 | 0.0065 | 3.2E-15 |
| 135 | rs11042902 | 11p15.4 | 10,655,623 | T | 0.04 | 0.0066 | 3.8E-09 |
| 136 | rs7130870 | 11p13 | 36,344,202 | C | 0.04 | 0.0064 | 9.0E-09 |
| 137 | rs714417 | 11p11.2 | 45,247,176 | C | 0.04 | 0.0065 | 7.5E-08 |
| 138 | rs174551 | 11q12.2 | 61,573,684 | T | 0.05 | 0.0064 | 1.0E-13 |
| 139 | rs479844 | 11q13.1 | 65,551,957 | G | 0.05 | 0.0060 | 1.0E-18 |
| 140 | rs7936323 | 11q13.5 | 76,293,758 | A | 0.11 | 0.0060 | 5.9E-74 |
| 141 | rs56324483 | 11q21 | 95,424,511 | G | 0.04 | 0.0090 | 4.6E-05 |
| 142 | rs1784776 | 11q23.1 | 111,472,392 | T | 0.04 | 0.0066 | 1.5E-10 |
| 143 | rs12365699 | 11q23.3 | 118,743,286 | G | 0.07 | 0.0081 | 1.7E-17 |
| 144 | rs55836957 | 11q24.3 | 128,168,631 | C | 0.05 | 0.0073 | 1.1E-11 |
| 145 | rs56389811 | 12q13.11 | 48,205,358 | C | 0.04 | 0.0071 | 5.8E-09 |
| 146 | rs3759129 | 12q13.12 | 50,354,437 | C | 0.04 | 0.0076 | 3.0E-06 |
| 147 | rs1689510 | 12q13.2 | 56,396,768 | C | 0.07 | 0.0064 | 2.8E-27 |
| 148 | rs167769 | 12q13.3 | 57,503,775 | T | 0.07 | 0.0062 | 8.6E-31 |
| 149 | rs1051334 | 12q21.1 | 71,523,134 | A | 0.04 | 0.0061 | 3.4E-09 |
| 150 | rs150449584 | 12q21.31 | 85,793,022 | C | 0.10 | 0.0251 | 1.1E-04 |
| 151 | rs12303699 | 12q22 | 94,582,336 | A | 0.04 | 0.0062 | 1.9E-09 |
| 152 | rs653178 | 12q24.12 | 112,007,756 | T | 0.03 | 0.0061 | 9.1E-08 |
| 153 | rs625228 | 12q24.31 | 121,278,266 | A | 0.04 | 0.0060 | 9.5E-11 |
| 154 | rs7132277 | 12q24.31 | 123,593,382 | C | 0.04 | 0.0077 | 1.1E-06 |
| 155 | rs7323267 | 13q14.11 | 41,204,015 | C | 0.03 | 0.0075 | 4.8E-04 |
| 156 | rs61960013 | 13q14.11 | 44,490,181 | G | 0.04 | 0.0074 | 7.0E-09 |
| 157 | rs981625 | 13q22.1 | 74,039,935 | G | 0.08 | 0.0124 | 7.5E-10 |
| 158 | rs7987173 | 13q32.3 | 100,073,342 | C | 0.05 | 0.0061 | 3.5E-16 |
| 159 | rs10162476 | 14q13.1 | 34,144,433 | G | 0.02 | 0.0061 | 1.2E-04 |
| 160 | rs34332679 | 14q13.2 | 35,881,966 | GA | 0.04 | 0.0063 | 9.9E-11 |
| 161 | rs34307686 | 14q13.3 | 37,651,998 | CTG | 0.02 | 0.0068 | 2.2E-03 |
| 162 | rs3751289 | 14q23.1 | 61,983,943 | G | 0.04 | 0.0073 | 9.8E-08 |
| 163 | rs911263 | 14q24.1 | 68,753,593 | C | 0.05 | 0.0067 | 9.2E-16 |
| 164 | rs888414 | 14q24.3 | 75,104,905 | A | 0.02 | 0.0062 | 9.7E-05 |
| 165 | rs10131197 | 14q32.12 | 93,015,394 | G | 0.04 | 0.0064 | 2.6E-10 |
| 166 | rs59457020 | 14q32.31 | 103,190,843 | G | 0.05 | 0.0092 | 4.5E-09 |
| 167 | rs1942 | 15q15.1 | 41,774,423 | G | 0.04 | 0.0060 | 3.7E-11 |
| 168 | rs11071559 | 15q22.2 | 61,069,988 | C | 0.09 | 0.0087 | 2.4E-27 |
| 169 | rs17293632 | 15q22.33 | 67,442,596 | T | 0.11 | 0.0071 | 5.7E-57 |
| 170 | rs11259930 | 15q25.2 | 84,577,350 | A | 0.03 | 0.0061 | 8.5E-09 |
| 171 | rs8029440 | 15q26.1 | 91,409,514 | G | 0.03 | 0.0063 | 8.8E-07 |
| 172 | rs11645975 | 16p13.3 | 3,749,397 | G | 0.03 | 0.0069 | 3.5E-05 |
| 173 | rs12935657 | 16p13.13 | 11,219,041 | G | 0.09 | 0.0070 | 3.8E-41 |
| 174 | rs3024655 | 16p12.1 | 27,369,502 | G | 0.12 | 0.0122 | 1.4E-21 |
| 175 | rs2066844 | 16q12.1 | 50,745,926 | T | 0.09 | 0.0145 | 3.2E-09 |
| 176 | rs223819 | 16q13 | 57,394,862 | T | 0.05 | 0.0112 | 2.3E-06 |
| 177 | rs71368508 | 17p13.2 | 4,521,473 | C | 0.13 | 0.0213 | 6.8E-10 |
| 178 | rs72842819 | 17p13.1 | 7,328,821 | C | 0.06 | 0.0094 | 3.2E-09 |
| 179 | rs750065349 | 17p12 | 12,193,443 | GC | 0.04 | 0.0060 | 8.6E-10 |
| 180 | rs2305479 | 17q12 | 38,062,217 | C | 0.11 | 0.0060 | 2.9E-69 |
| 181 | rs2006141 | 17q21.2 | 40,679,718 | T | 0.04 | 0.0067 | 8.2E-08 |
| 182 | rs7224548 | 17q21.31 | 43,337,136 | G | 0.05 | 0.0066 | 1.1E-13 |
| 183 | rs72833417 | 17q21.32 | 45,873,049 | T | 0.06 | 0.0093 | 3.3E-12 |
| 184 | rs17637472 | 17q21.33 | 47,461,433 | A | 0.06 | 0.0063 | 7.7E-20 |
| 185 | rs1991401 | 17q23.3 | 62,502,435 | A | 0.04 | 0.0065 | 1.1E-08 |
| 186 | rs111365807 | 17q25.1 | 73,825,463 | C | 0.05 | 0.0093 | 1.2E-07 |
| 187 | rs76848919 | 17q25.3 | 76,352,554 | C | 0.04 | 0.0075 | 5.6E-09 |
| 188 | rs12956924 | 18q21.1 | 46,451,146 | A | 0.04 | 0.0065 | 3.7E-08 |
| 189 | rs12453988 | 18q21.2 | 48,581,918 | T | 0.03 | 0.0062 | 1.7E-07 |
| 190 | rs3730775 | 18q21.2 | 51,813,966 | T | 0.04 | 0.0061 | 3.3E-10 |
| 191 | rs3826620 | 18q21.33 | 60,021,504 | G | 0.04 | 0.0066 | 1.3E-09 |
| 192 | rs12964116 | 18q21.33 | 61,442,619 | G | 0.10 | 0.0160 | 2.6E-09 |
| 193 | rs10853952 | 19p13.3 | 1,163,934 | T | 0.03 | 0.0065 | 1.4E-06 |
| 194 | rs117552144 | 19p13.3 | 3,136,091 | T | 0.09 | 0.0130 | 3.6E-11 |
| 195 | rs10420217 | 19p13.3 | 4,355,871 | C | 0.04 | 0.0067 | 2.0E-08 |
| 196 | rs755023315 | 19p13.3 | 6,579,029 | G | 0.04 | 0.0068 | 2.7E-09 |
| 197 | rs10416530 | 19p13.2 | 9,129,660 | C | 0.04 | 0.0064 | 6.0E-11 |
| 198 | rs34006614 | 19p13.11 | 16,442,782 | T | 0.03 | 0.0064 | 1.1E-07 |
| 199 | rs118013485 | 19q13.11 | 33,726,577 | G | 0.14 | 0.0123 | 3.3E-29 |
| 200 | rs143432496 | 19q13.32 | 45,252,714 | A | 0.05 | 0.0068 | 2.3E-11 |
| 201 | rs8103278 | 19q13.32 | 46,370,381 | G | 0.04 | 0.0063 | 1.2E-08 |
| 202 | rs11670020 | 19q13.41 | 52,314,161 | G | 0.04 | 0.0086 | 1.7E-06 |
| 203 | rs8125525 | 20q13.12 | 45,681,788 | C | 0.04 | 0.0069 | 1.7E-07 |
| 204 | rs6021270 | 20q13.2 | 50,141,264 | T | 0.07 | 0.0125 | 1.0E-08 |
| 205 | rs2766667 | 20q13.2 | 52,172,404 | T | 0.05 | 0.0069 | 1.9E-11 |
| 206 | rs6011033 | 20q13.33 | 62,322,699 | G | 0.05 | 0.0071 | 9.7E-13 |
| 207 | rs1736147 | 21q21.1 | 16,813,053 | G | 0.03 | 0.0061 | 1.1E-07 |
| 208 | rs2242900 | 21q22.12 | 36,453,837 | G | 0.07 | 0.0087 | 1.1E-15 |
| 209 | rs34846236 | 21q22.2 | 41,049,344 | G | 0.03 | 0.0061 | 5.3E-06 |
| 210 | rs12626388 | 21q22.3 | 43,856,593 | T | 0.04 | 0.0085 | 2.4E-06 |
| 211 | rs228953 | 22q12.3 | 37,531,436 | G | 0.03 | 0.0061 | 8.1E-06 |
| 212 | rs201267172 | 22q13.2 | 41,918,653 | G | 0.05 | 0.0076 | 1.3E-12 |

*Abbreviations: SNPs: Single-nucleotide Polymorphisms; CHR, Chromosome; EA, Effect Allele; SE, Standard Error.

**Table S2. Baseline characteristics of 307,872 UK biobank participants according to the serum vitamin D levels.**

|  | **Serum Vitamin D (nmol/L)** | | | **Total**  **(n=307,872)** | ***P*** |
| --- | --- | --- | --- | --- | --- |
|  | **< 25 (n=35,495)** | **25~50 (n=127,446)** | **> 50 (n=144,931)** |  |  |
| Asthma, N (%) | 808 (2.3) | 2,629 (2.1) | 2,706 (1.9) | 6,143 (2.0) | < 0.001 |
| Age (year), mean ± SD | 55.29 ± 8.05 | 56.35 ± 8.05 | 57.31 ± 7.98 | 56.68 ± 8.04 | < 0.001 |
| Age category (years), N (%) |  |  |  |  | < 0.001 |
| < 50 | 11,080 (31.2) | 34,249 (26.9) | 33,331 (23.0) | 78,660 (25.5) |  |
| 50-59 | 11,706 (33.0) | 39,776 (31.2) | 42,087 (29.0) | 93,569 (30.4) |  |
| > 60 | 12,709 (35.8) | 53,421 (41.9) | 69,513 (48.0) | 135,643 (44.1) |  |
| Sex, N (%) |  |  |  |  | 0.063 |
| Female | 19,220 (54.1) | 68,355 (53.6) | 78,315 (54.0) | 165,890 (53.9) |  |
| Male | 16,275 (45.9) | 59,091 (46.4) | 66,616 (46.0) | 141,982 (46.1) |  |
| Race, N (%) |  |  |  |  | < 0.001 |
| White | 46 (0.1) | 128 (0.1) | 95 (0.1) | 269 (0.1) |  |
| British | 32,659 (92.0) | 118,692 (93.1) | 137,169 (94.6) | 288,520 (93.7) |  |
| Irish | 1,157 (3.3) | 3,734 (2.9) | 3,660 (2.5) | 8,551 (2.8) |  |
| Other white background | 1,633 (4.6) | 4,892 (3.8) | 4,007 (2.8) | 10,532 (3.4) |  |
| BMI (kg/m^2^), mean ± SD | 28.40 ± 5.63 | 27.73 ± 4.82 | 26.56 ± 4.11 | 27.26 ± 4.66 | < 0.001 |
| BMI category, N (%) |  |  |  |  | < 0.001 |
| Normal (< 25) | 10,342 (29.3) | 38,650 (30.4) | 55,571 (38.4) | 104,563 (34.1) |  |
| Overweight (25~30) | 13,399 (38.0) | 54,336 (42.8) | 63,494 (43.9) | 131,229 (42.7) |  |
| Obesity (30~) | 11,560 (32.7) | 34,076 (26.8) | 25,546 (17.7) | 71,182 (23.2) |  |
| Average Total Household Income Before Tax (€), N (%) |  |  |  |  | < 0.001 |
| Less than 18,000 | 7,702 (21.7) | 22,293 (17.5) | 24,114 (16.6) | 54,109 (17.6) |  |
| 18,000 to 30,999 | 7,460 (21.0) | 27,690 (21.7) | 32,683 (22.6) | 67,833 (22.0) |  |
| 31,000 to 51,999 | 8,227 (23.2) | 30,376 (23.8) | 33,688 (23.2) | 72,291 (23.5) |  |
| 52,000 to 100,000 | 6,541 (18.4) | 24,862 (19.5) | 27,032 (18.7) | 58,435 (19.0) |  |
| Greater than 100,000 | 1,525 (4.3) | 6,595 (5.2) | 7,616 (5.3) | 15,736 (5.1) |  |
| Unknown | 4,040 (11.4) | 15,630 (12.3) | 19,788 (13.7) | 39,468 (12.8) |  |
| Missing data | 150 (0.4) | 276 (0.2) | 197 (0.1) | 623 (0.2) |  |
| Education, N (%) |  |  |  |  | < 0.001 |
| College or University Degree | 12,558 (35.4) | 43,435 (34.1) | 43,435 (30.0) | 99,428 (32.3) |  |
| Professional qualifications | 3,929 (11.1) | 14,698 (11.5) | 17,615 (12.2) | 36,242 (11.8) |  |
| A Levels/AS Levels or Equivalent | 4,183 (11.8) | 14,576 (11.4) | 16,146 (11.1) | 34,905 (11.3) |  |
| O Levels/GCSEs or Equivalent | 8,792 (24.8) | 33,492 (26.3) | 41,564 (28.7) | 83,848 (27.2) |  |
| None of the above | 6,033 (17.0) | 21,245 (16.7) | 26,171 (18.1) | 53,449 (17.4) |  |
| Smoking status, N (%) |  |  |  |  | < 0.001 |
| Never | 17,852 (50.3) | 68,854 (54.0) | 78,638 (54.3) | 165,344 (53.7) |  |
| Previous | 11,495 (32.4) | 44,713 (35.1) | 53,706 (37.1) | 109,914 (35.7) |  |
| Current | 6,044 (17.0) | 13,564 (10.6) | 12,164 (8.4) | 31,772 (10.3) |  |
| None of the above | 104 (0.3) | 315 (0.2) | 423 (0.3) | 842 (0.3) |  |
| Vitamin D supplements, N (%) | 182 (0.5) | 1,389 (1.1) | 3,836 (2.6) | 5,407 (1.8) | < 0.001 |
| Sleep duration, N (%) |  |  |  |  | < 0.001 |
| Ideal | 23,148 (65.2) | 87,905 (69.0) | 102,215 (70.5) | 94,604 (30.7) |  |
| Poor | 12,347 (34.8) | 39,541 (31.0) | 42,716 (29.5) | 213,268 (69.3) |  |
| Chronotype, N (%) |  |  |  |  | < 0.001 |
| Ideal | 20,085 (56.6) | 78,790 (61.8) | 95,075 (65.6) | 193,950 (63.0) |  |
| Poor | 15,410 (43.4) | 48,656 (38.2) | 49,856 (34.4) | 113,922 (37.0) |  |
| Insomnia, N (%) |  |  |  |  | 0.001 |
| Ideal | 8,677 (24.4) | 31,962 (25.1) | 35,530 (24.5) | 76,169 (24.7) |  |
| Poor | 26,818 (75.6) | 95,484 (74.9) | 109,401 (75.5) | 231,703 (75.3) |  |
| Snoring, N (%) |  |  |  |  | < 0.001 |
| Ideal | 22,290 (62.8) | 79,092 (62.1) | 92,685 (64.0) | 194,067 (63.0) |  |
| Poor | 13,205 (37.2) | 48,354 (37.9) | 52,246 (36.0) | 113,805 (37.0) |  |
| Daytime sleepiness, N (%) |  |  |  |  | < 0.001 |
| Ideal | 34,361 (96.8) | 124,150 (97.4) | 141,636 (97.7) | 300,147 (97.5) |  |
| Poor | 1,134 (3.2) | 3,296 (2.6) | 3,295 (2.3) | 7,725 (2.5) |  |
| Healthy sleep score, N (%) |  |  |  |  | < 0.001 |
| 0 | 127 (0.4) | 288 (0.2) | 205 (0.1) | 620 (0.2) |  |
| 1 | 19,24 (5.4) | 5,250 (4.1) | 4,904 (3.4) | 12,078 (3.9) |  |
| 2 | 7,853 (22.1) | 25,642 (20.1) | 2,238 (18.1) | 59,733 (19.4) |  |
| 3 | 13,628 (38.4) | 48,735 (38.2) | 55,798 (38.5) | 118,161 (38.4) |  |
| 4 | 9,768 (27.5) | 38,495 (30.2) | 46,563 (32.1) | 94,826 (30.8) |  |
| 5 | 2,195 (6.2) | 9,036 (7.1) | 11,223 (7.7) | 22,454 (7.3) |  |
| Sleep patterns, N (%) |  |  |  |  | < 0.001 |
| Healthy | 11,963 (33.7) | 47,531 (37.3) | 57,786 (39.9) | 117,280 (38.1) |  |
| Intermediate | 21,481 (60.5) | 74,377 (58.4) | 82,036 (56.6) | 177,894 (57.8) |  |
| Poor | 2,051 (5.8) | 5,538 (4.3) | 177,894 (3.5) | 12,698 (4.1) |  |

^a^Abbreviations: SD: Standard Deviation.

**Table S3. Sensitivity analysis for association between serum vitamin D levels and the risk of incident asthma after excluding incident events occurred within the third year.**

| **Serum vitamin D levels** | **Case /N** | **Model 1^*^** | |  | **Model 2^‡^** | |  | **Model 3^§^** | |
| --- | --- | --- | --- | --- | --- | --- | --- | --- | --- |
|  |  | **HR (95% CI)** | **P** |  | **HR (95% CI)** | **P** |  | **HR (95% CI)** | **P** |
| Deficient  (< 25 nmol/L) | 551/35,238 | Ref. | - |  | Ref. | - |  | Ref. | - |
| Insufficient  (25~50 nmol/L) | 1,762/126,579 | 0.890 (0.809, 0.979) | 0.017 |  | 0.872 (0.793, 0.960) | 0.005 |  | 0.935 (0.849, 1.030) | 0.175 |
| Optimal  (> 50 nmol/L) | 1,836/144,061 | 0.814 (0.740, 0.895) | < 0.001 |  | 0.783 (0.712, 0.862) | < 0.001 |  | 0.891 (0.807, 0.984) | 0.023 |
| P_trend_ |  | < 0.001 |  |  | < 0.001 |  |  | 0.062 |  |

*Model 1: Crude.

‡Model 2: Adjusted for age, sex, race.

§Model 3: Adjusted for age, sex, race, BMI, income, education, smoking status, assessment center and vitamin D supplements.

**Table S4. Sensitivity analysis for association between serum vitamin D levels and the risk of incident asthma after performing multiple imputation for missing covariates.**

| **Serum vitamin D levels** | **Case /N** | **Model 1^*^** | |  | **Model 2^‡^** | |  | **Model 3^§^** | |
| --- | --- | --- | --- | --- | --- | --- | --- | --- | --- |
|  |  | **HR (95% CI)** | **P** |  | **HR (95% CI)** | **P** |  | **HR (95% CI)** | **P** |
| Deficient  (< 25 nmol/L) | 808/35,495 | Ref. | - |  | Ref. | - |  | Ref. | - |
| Insufficient  (25~50 nmol/L) | 2,629/127,446 | 0.905 (0.837, 0.980) | 0.014 |  | 0.887 (0.820, 0.960) | 0.003 |  | 0.958 (0.885, 1.038) | 0.297 |
| Optimal  (> 50 nmol/L) | 2,706/144,931 | 0.819 (0.757, 0.886) | < 0.001 |  | 0.786 (0.726, 0.851) | < 0.001 |  | 0.916 (0.845, 0.994) | 0.035 |
| P_trend_ |  | < 0.001 |  |  | < 0.001 |  |  | 0.071 |  |

*Model 1: Crude.

‡Model 2: Adjusted for age, sex, race.

§Model 3: Adjusted for age, sex, race, BMI, income, education, smoking status, assessment center and vitamin D supplements.

**Table S5. Interaction effect between serum vitamin D levels, genetic risk and sleep patterns.**

|  | **Coeffect** | ***P*** |
| --- | --- | --- |
| Serum vitamin D × Intermediate sleep | 0.926 (0.895, 0.958) | < 0.001 |
| Serum vitamin D × Poor sleep | 0.833 (0.738, 0.941) | < 0.001 |
| Serum vitamin D × Intermediate genetic risk | 0.910 (0.870, 0.951) | < 0.001 |
| Serum vitamin D × High genetic risk | 0.952 (0.912, 0.995) | 0.029 |

*References were healthy sleep and low genetic risk.

**Table S6. Association between sleep patterns with risk of asthma.**

|  | **Case /N** | **Model 1^*^** | |  | **Model 2^‡^** | |  | **Model 3^§^** | |
| --- | --- | --- | --- | --- | --- | --- | --- | --- | --- |
|  |  | **HR (95% CI)** | **P** |  | **HR (95% CI)** | **P** |  | **HR (95% CI)** | **P** |
| Healthy Sleep Score |  |  |  |  |  |  |  |  |  |
| 0 | 23/597 | Ref. | - |  | Ref. | - |  | Ref. | - |
| 1 | 335/11,743 | 0.743 (0.487, 1.134) | 0.168 |  | 0.739 (0.484, 1.128) | 0.161 |  | 0.824 (0540, 1.257) | 0.369 |
| 2 | 1,456/58,277 | 0.652 (0.432, 0.984) | 0.042 |  | 0.640 (0.424, 0.966) | 0.034 |  | 0.771 (0.510, 1.164) | 0.215 |
| 3 | 2,411/115,750 | 0.544 (0.361, 0.821) | 0.004 |  | 0.528(0.350, 0.796) | 0.002 |  | 0.682 (0.452, 1.028) | 0.068 |
| 4 | 1,611/93,215 | 0.453 (0.300, 0.683) | < 0.001 |  | 0.439 (0.291, 0.663) | < 0.001 |  | 0.061 (0.398, 0.908) | 0.015 |
| 5 | 307/22,147 | 0.364 (0.238, 0.555) | < 0.001 |  | 0.366 (0.240, 0.559) | < 0.001 |  | 0.522 (0.341, 0.798) | 0.003 |
| P_trend_ |  | < 0.001 |  |  | < 0.001 |  |  | < 0.001 |  |
| Sleep patterns |  |  |  |  |  |  |  |  |  |
| Healthy | 1,918/115,362 | Ref. | - |  | Ref. | - |  | Ref. | - |
| Intermediate | 3,867/174,027 | 1.332 (1.261, 1.407) | < 0.001 |  | 1.329 (1.258, 1.403) | < 0.001 |  | 1.213 (1.147, 1.282) | < 0.001 |
| Poor | 358/12,340 | 1.735 (1.550, 1.942) | < 0.001 |  | 1.766 (1.577,1.977) | < 0.001 |  | 1.415 (1.262, 1.587) | < 0.001 |
| P_trend_ |  | < 0.001 |  |  | <0.001 |  |  | < 0.001 |  |

*Model 1: Crude.

‡Model 2: Adjusted for age, sex, race.

§Model 3: Adjusted for age, sex, race, BMI, income, education, smoking status, assessment center and vitamin D supplements.

**Table S7. Association between genetic risk score with risk of asthma.**

| **Quintiles of PRS** | **Case /N** | **Model 1^*^** | |  | **Model 2^‡^** | |  | **Model 3^§^** | |
| --- | --- | --- | --- | --- | --- | --- | --- | --- | --- |
|  |  | **HR (95% CI)** | **P** |  | **HR (95% CI)** | **P** |  | **HR (95% CI)** | **P** |
| Continuous | 6,143/307,872 | 1.566 (1.481, 1.656) | < 0.001 |  | 1.562 (1.477, 1.652) | < 0.001 |  | 0.965 (0.940, 0.991) | 0.009 |
| Categorical |  |  |  |  |  |  |  |  |  |
| low | 2,600/153,936 | Ref. | - |  | Ref. | - |  | Ref. | - |
| high | 3,543/153,936 | 1.366 (1.299, 1.437) | < 0.001 |  | 1.364 (1.297, 1.435) | < 0.001 |  | 1.365 (1.297, 1.436) | < 0.001 |
| Categorical |  |  |  |  |  |  |  |  |  |
| low | 1,604/102,614 | Ref. | - |  | Ref. | - |  | Ref. | - |
| intermediate | 2,034/102,614 | 1.270 (1.190, 1.356) | < 0.001 |  | 1.270 (1.189, 1.356) | < 0.001 |  | 1.268 (1.187, 1.354) | < 0.001 |
| high | 2,505/102,644 | 1.567 (1.472, 1.669) | < 0.001 |  | 1.564 (1.469, 1.665) | < 0.001 |  | 1.566 (1.470, 1.667) | < 0.001 |
| P_trend_ |  | < 0.001 |  |  | < 0.001 |  |  | < 0.001 |  |

*Model 1: Crude.

‡Model 2: Adjusted for age, sex, race.

§Model 3: Adjusted for age, sex, race, BMI, income, education, smoking status, assessment center and vitamin D supplements.
